# Case of Diabetes, Successfully Treated

**Published:** 1829-04

**Authors:** G. T. Burnett


### DIABETES.

*Case of Diabetes, successfully treated.* By G. T. Burnett, Esq.

December 31st, 1823.—Consulted by Mr. J. H., aged forty-seven, who states that for some time past, (at least for several, perhaps for many months,) he has been in not quite such good health as he formerly enjoyed. Of the change he himself is conscious, and his family have remarked his altered look; but, as no pain is suffered, and as he is able to transact his usual business, (wholesale spirit merchant,) he has hitherto not sought medical advice. He has lately lost much flesh, but, as his appetite continued good, even better than ordinary, this wasting was at first attributed to over-exertion and fatigue in business.

The present complaints are chiefly thirst, which has been gradually increasing, and is now constant and distressing, the mouth being ever dry or clammy, requiring to be continually moistened. During the day and in the night he drinks two or three pints of thin gruel or water. His countenance has lost its natural healthy colour and roundness, and has assumed a pale and sharp appearance; the lips are dry and cracking, and the whole skin extremely harsh. Perspirations, which used to be considerable on exertion, entirely stopped, and the urine greatly increased in quantity. These symptoms have been gradually coming on for a considerable time, yet they remained unnoticed; and it was not until the frequent desire during the day, and the necessity of rising several times during the night, to discharge the water, became very troublesome and disturbed his rest, that they excited attention. He now gets up five or six times during the night, and passes in general seven or eight pints (often more) of urine; and, in the day, he says he can scarcely walk half a mile without stopping for the same purpose; and he observes that, wherever the urine splashes, it leaves a whitish sticky spot, of which he shows many at the lower part of his trowsers. Pulse frequent, not full; tongue furred, white at the edges, brownish in the centre; bowels open daily; urine of a pale straw colour, bitter sweetish taste, and perfumy, does not deposit any sediment on cooling.

Purges ordered, with sudorific medicines; spirits and vegetable food prohibited; animal diet strictly enjoined.

These directions were partially complied with for about a week or ten days; but, being impatient of restraint, the thirst being a little relieved, appetite great, strength little impaired, and business urgent, they were then neglected.

March 10th. 1824.—Since the last report the disease has been gradually establishing itself, and the symptoms becoming daily more urgent: repetition would be useless; they are the same as above noted, only increased in severity. The emaciation is very considerable, so much so that his family say that they "can see him fall away." His clothes, which used to fit tightly, are much too loose, and hang in folds. Some degree of lassitude has shown itself. Appetite truly voracious., Having, in answer to some inquiries, stated his complaint to be diabetes, and Mrs. H. studying Buchan, they are now much alarmed, and willing to submit to any treatment.

Former directions repeated, and former treatment resumed.

March 11th, 12th, 13th, and 14th.—No amendment. Thirst quite as great, urine as copious; skin very dry and often hot, but not the slightest moisture.—Let the sudorific medicines (viz. Pulv. Ipecac. c. gr. xij.; Pulv. Antim. gr. v.; Pulv. Acaciæ ℈ss.) be taken at seven, and again at ten each evening; and, an hour after each powder, Liq. Amm. Acet. ℥i.; Mist. Camph. ℥ss. Let the feet and legs be placed in hot water, the bed warmed, and abundance of warm milk-and-water given him to drink. Let him keep in a warm room during the day, and take saline sudorifics, and Haust. Sennæ every alternate morning.

18th.—A slight degree of moisture appeared on the chest last night, which has raised hope and encourages perseverance. Other things as before.

22d.—Perspirations have slightly increased each night; thirst perhaps not quite so troublesome; lips parched and cracked; urine still as copious, sweet both to taste and smell: on evaporation, it affords a large quantity of bitter sweet extractive matter, of a brown colour; upwards of one ounce from a pint of urine.

23d.—Sickness came on last night, and the medicines rejected.—Let only one powder and draught be taken each night, and the laxative medicine in the morning.

25th.—Perspiration lessened; other symptoms as before. —Let the double doses be again exhibited for one night.

27th.—Perspirations very free; tongue less furred; urine less abundant, but still unnaturally copious. Passed last night without being once disturbed, which has not happened for many months before. The lips are less parched, the thirst much less, and the countenance improving, having lost some of its anxiety and sharpness.

29th.—All going on favorably; urine reduced to its natural quantity, or nearly so; but Mr. H. presumes too much on his recovery, and appears anxious to discontinue the medicines.—The ordinary cautions given, and warm sea-bathing recommended during the summer.

Health continued good for several months: cautious living, sea air, bathing, &c. seemed to have completely re-established him. When I occasionally met him, he appeared delighted with the change, but showed evidently that he thought he had been the best judge as to the period of discontinuing the medicine.

October.—Having been to two public dinners within a week, and indulged rather too freely in the pleasures of the table, I was applied to on the 14th. He was much dejected by the return of his old symptoms, though in a milder form, and, although the harshness of skin, thirst, and abundance of urine had not returned above a week, the change in his appearance was surprising; the sharpness of the features, and sallowness and anxiety, were so great. The treatment before so successfully pursued was again resorted to: in less than a week the diseased actions were subdued, and within a fortnight all unpleasant symptoms were removed.

January 9th, 1825.—Having neglected to observe due caution as to diet, exercise, and exposure, the tendency to diabetes has again shown itself. The flesh which had been gained during the summer and autumn is now diminishing, and that very rapidly. The symptoms again yielded to the treatment previously detailed: within a week they were much abated, and by February 2d the sudorifics were discontinued, and mineral acid prescribed for a fortnight.

In May, from the nature of his business, having been again obliged to attend some public dinners, he had another return of the diabetic symptoms, and under similar treatment they were again removed.

November 17th.—I was again sent to; for his frequent recovery seemed now to have rendered him less cautious. To endeavour to impress him with the necessity of attending to diet, and abstaining from excess in liquors, an eminent physician was consulted, and, with the previous history of its efficacy, similar treatment was adopted, with a similar result.

In the following March (1826,) another slight relapse followed some accidental irregularity; and, to wean him from public dinners, I recommended him to take a country house, and amuse himself in gardening; which he did, and since then, with caution on his own part, aided by the watchful care of an anxious wife, he has avoided any further relapse; his health has been uniformly good, and when I saw him (March 9th, 1829,) he was "as hale and hearty" as ever he felt in his life; and, excepting slight cough, and that only *slight* and occasional, he has had no illness, and has taken no medicine, save domestic aperients, for the last three years.

The observations on the foregoing case might perhaps be many, but they shall be few. To speculate on single instances, either of failure or success, is futile, and to generalize from such meagre experience would be absurd; yet, with respect to so interesting a disease, the pathology of which is so obscure, and the treatment of which is so undecided, each additional fact must have its value, and, as many cases of a complaint so comparatively rare cannot be expected to fall under the notice of the same individual, it becomes the duty of every one to cast his mite into the treasury of public knowledge.

I have seen several cases of diabetes in hospital and other practice; I have watched them with considerable care; I have inquired into their history, examined their symptoms, noted their treatment, and anxiously waited for the result. I have seen most of the ordinary modes of treatment prescribed and persevered in for a considerable period, sometimes with temporary, but never with permanent relief; for there was not one that did not eventuate in the despair, either of the patient or the prescriber, or of both.

There is nothing novel in the treatment which cured the case that I have related, and there is but one practical point that it suggests: viz. that, although diaphoretics are frequently prescribed, they are seldom effectively administered; for it is a disease on which so little impression is made, and so little relief obtained, that hope becomes languid, and neglect supervenes. Furthermore, in the early stages of diabetes, in which perhaps it alone is curable, the difficulty is great to persuade a person, in some measure able to go through the duties of his calling, and whose appetite is good, to refrain from accustomed indulgences, and to persevere with an irksome treatment: hence remedies are too often imperfectly employed, and pursued for a very inadequate period.

*Great Marylebone street;*
*March* 9*th*, 1829.

